# Supplementary material for: Nanoscale dynamics of cellulose digestion by the cellobiohydrolase TrCel7A
Source: J Biol Chem. 2021 Jul 31;297(3):101029. doi: 10.1016/j.jbc.2021.101029 (PMC8390518; doi:10.1016/j.jbc.2021.101029)
Supplement: Figures S1–S5 [file mmc1.docx]

**Haviland et al: Nanoscale Dynamics of Cellulose Digestion by the Cellobiohydrolase *Tr*Cel7A**

**Supplemental Figures:**


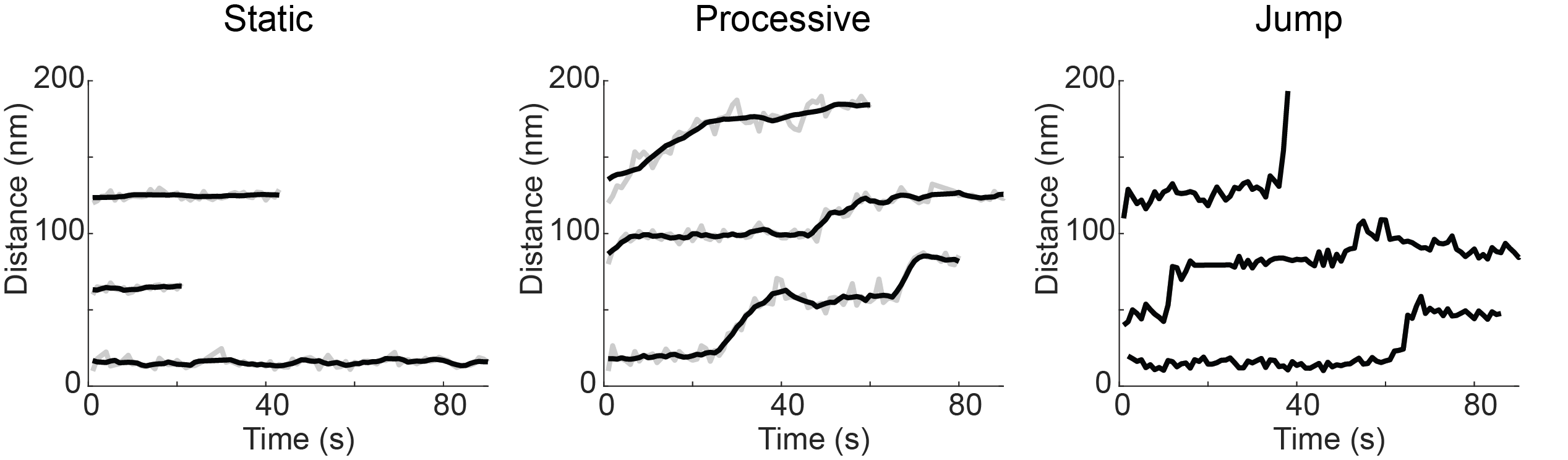


**Figure S1 (Related to Figure 3):** Single-molecule trajectories showing exemplary tracks for the three behaviors exhibited by TrCel7a on acetobacter cellulose. For Static and Processive tracks, the light grey lines plots represent the raw, drift-corrected position data at 1 frame/s, and bold black lines represent 5 frame boxcar average. For Jump traces, bold black line are 1 frame/s data to emphasize the rapid displacements.


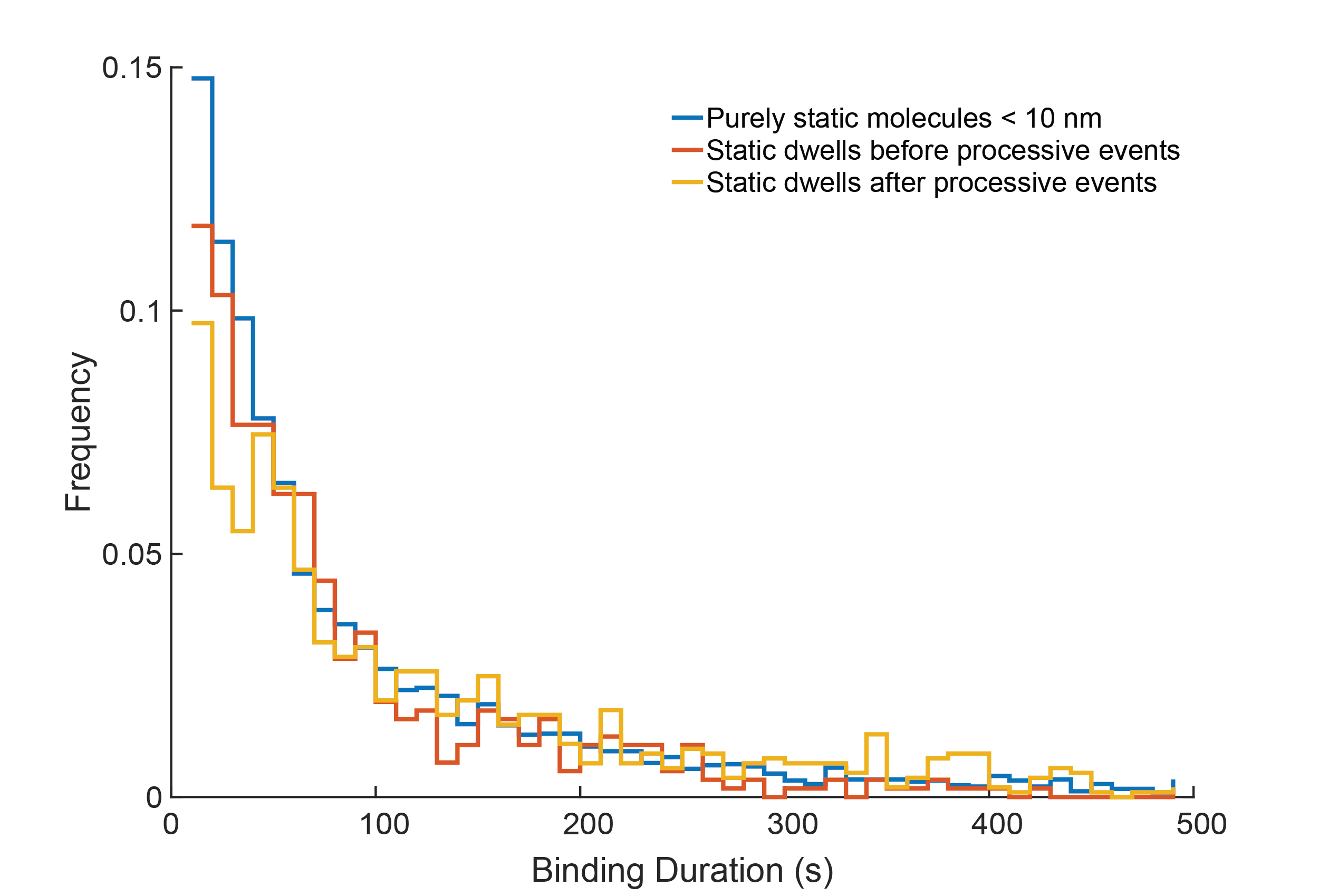


**Figure S2 (Related to Figure 4):** Data from Figure 3 plotted as a combined histogram to show similarity of static dwell times. Plot shows distribution of the dwell times for static segments before processive movement, static segments after processive movement, and purely static molecules that moved less than 10 nm from their original position. The bin size for the three traces are 10 seconds with a maximum time of 500 seconds, and all the values are normalized based on the number of data points in each population.


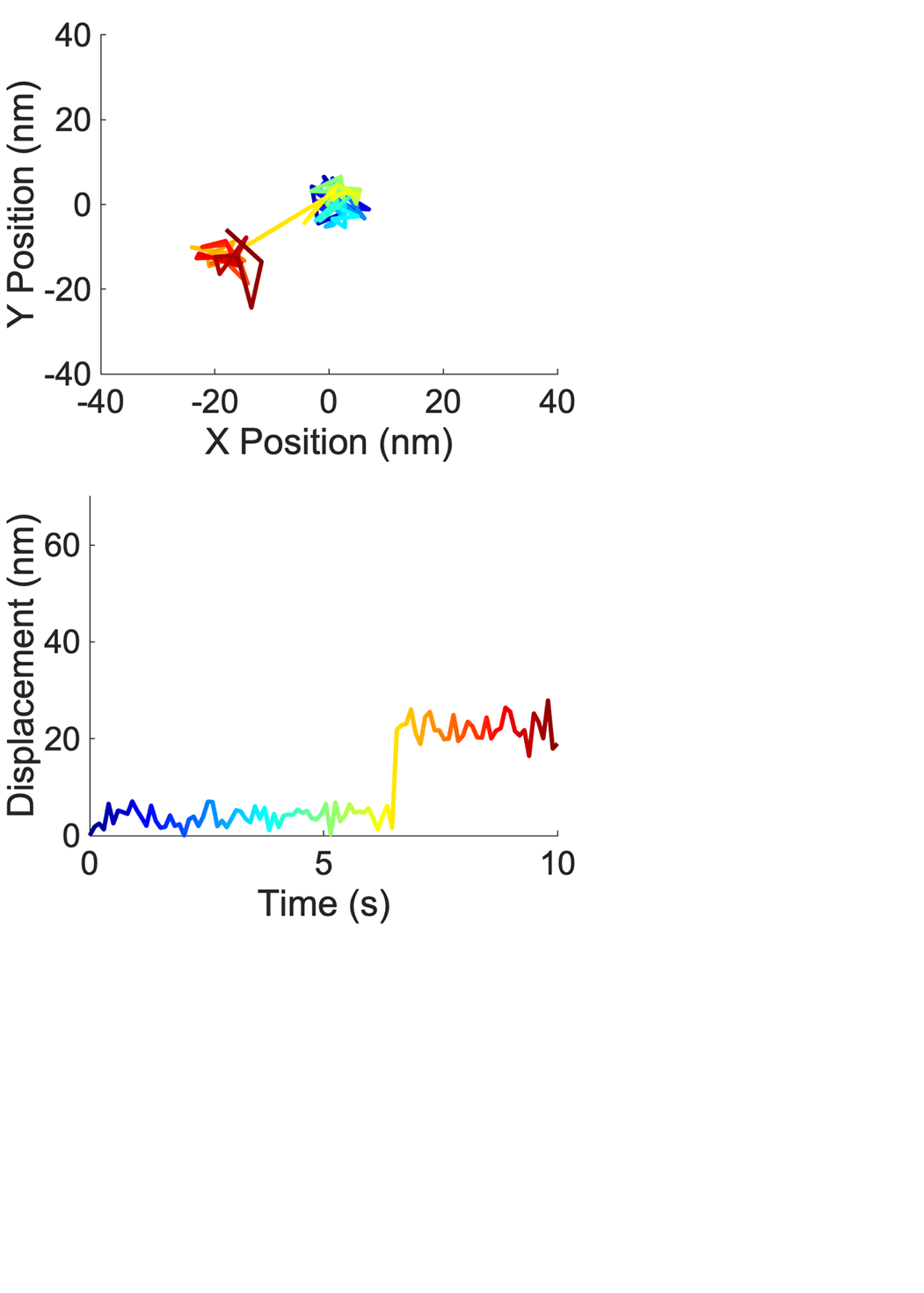


**Figure S3 (Related to Figure 3):** TrCel7a jumping behavior at 10 frames/s, to emphasize that the displacements are representative of fast diffusion through solution, rather than processive movement. The top plot is the X and Y position for the molecule and bottom plot is distance vs. time for the same trajectory. No data smoothing was performed. The possibility that these jumps were actually new particles landing while the existing particle dissociated was ruled out by the following argument. The entire screen was 6273 μm^2^, and so a 100 nm circle around any particle represents 5 x 10^-6^ of the total screen area. Particles landed at a rate of approximately 10/s, meaning that a new particle will land within 100 nm of an existing particle at the rate of 5 x 10^-5^ s^-1^. Particles dissociated at a rate of approximately 0.01 s^-1^, and so the probability of a particle dissociating in any given second is 0.01. Combining these values, simultaneous binding and unbinding events thus occur at a rate of 5 x 10^-7^ s^-1^ for any given particle. There were roughly 1000 particles on the screen at any given time, and the observation time was 1000 s. Hence, the expected number of “false jumps” is 0.5 (less than 1). From Table 1, jumps were observed in 7.3% of the 11,116 particles, meaning that 811 jumps were observed in 1000 s. Thus, we conclude that jumps represent the same particle dissociating and rapidly reassociating, and rule out the possibility that jumps represent simultaneous unbinding of one particle and rebinding of a different particle in the vicinity.


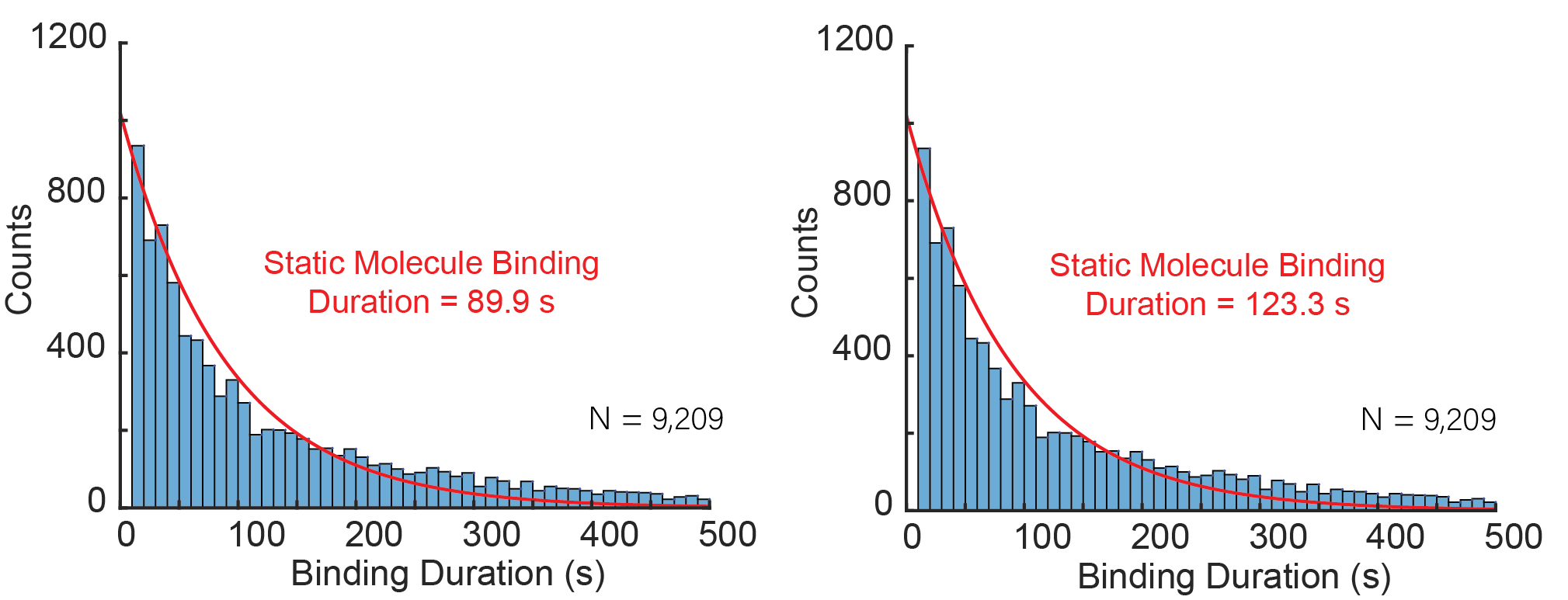


**Figure S4 (Related to Figure 4):** Histogram and average binding duration of all purely static molecules, including those with displacements > 10 nm and those containing noisy segments. Data were fit using MEMLET, as described in Material and Methods.


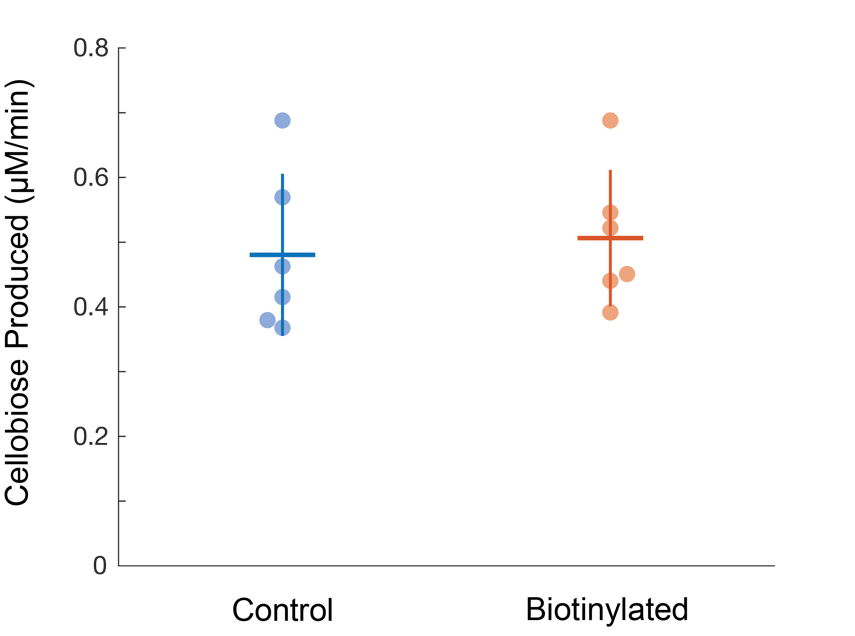


**Figure S5:** Reducing end assay results to determine the effect of biotinylation on the activity of TrCel7A enzymes. The control population displays the activity of wtCel7A, and the biotinylated population displays the activity of Cel7A enzymes where 95.9% of enzymes contain a biotin tag. Cellobiose produced by 200 nM TrCel7A on 4.5 mM acetobacter cellulose in 50 mM sodium acetate was measured over a span of 15 minutes.
